# Dry–Wet Cycle Fracture Performance of Recycled Concrete Sulfate Based on Computerized Tomographic Images

**DOI:** 10.3390/ma18173998

**Published:** 2025-08-26

**Authors:** Kefan Chen, Zhaoyi He, Hongxia Qiao

**Affiliations:** 1 School of Traffic and Transportation, Chongqing Jiaotong University, Chongqing 400074, China; zhaoyihe123zhao@163.com; 2 Chongqing Zhongjiao Renewable Resources Development Co., Ltd., Chongqing 401120, China; 3 School of Civil Engineering, Lanzhou University of Technology, Lanzhou 730050, China; qiaohongxia@lut.edu.cn

**Keywords:** recycled concrete, sulfate drying and wetting cycle, X-ray computed tomography, gray scale, stress intensity factor, fracture mechanics

## Abstract

To investigate the damage degradation of recycled concrete under mesoscale morphology and the critical expansion force of concrete cracking following sulfate wet–dry cycles, an experimental sulfate wet–dry cycle was designed. In situ scanning of recycled concrete was conducted using X-ray computed tomography (CT). Analysis of the CT images revealed the relationship between the gray scale changes and the sulfate salt wet–dry cycle, along with pore alterations and crack propagation in recycled concrete. A CT image analysis method based on grayscale inversion for crack propagation was developed. By integrating sulfate attack with fracture mechanics, this study explored the phenomenon of pore expansion in recycled concrete subjected to dry–wet cycling tests. The concrete fracture criterion provided the basis for determining the critical expansion force of recycled concrete after the wet–dry cycles. Results indicated that as the duration of sulfate wet–dry cycles increased, the gray scale first increased and then decreased. On the 40th day of the cycling test, the average grayscale value increased by 10.4%. The number of pores in recycled concrete continuously decreased, pore size diminished, and cracks appeared on the specimen’s weak surface. The use of gray scale changes to reveal the degradation of recycled concrete after sulfate wet–dry cycles proved to be both feasible and effective. As the length of the interface crack increased, the stress intensity factor at the crack tip also increased, while the critical expansion force decreased. Additionally, as the pore diameter increased, the stress intensity factor at the crack tip rose. The critical expansion force of a symmetric crack at the edge of a pore was 53 times greater than that of a single crack.

## 1. Introduction

Concrete is the most widely used construction material globally, yet its production imposes substantial pressure on natural resources and the environment. In particular, cement manufacturing is recognized as a major source of global CO_2_ emissions [1,2,3]. To achieve low-carbon development goals and transition towards sustainable construction, researchers are increasingly exploring resource-efficient and environmentally friendly material systems [4,5,6]. Among these, recycled aggregate concrete (RAC), derived from construction and demolition waste, has emerged as a promising approach to promoting circular economy and reducing the environmental footprint [7,8,9].

In recent years, the development of novel materials such as papercrete [10], mortar incorporating paper sludge ash [11], and 3D-printed recycled concrete [12] has expanded the functional scope and practical applications of recycled materials. However, RAC remains vulnerable to environmental deterioration during long-term service, among which sulfate attack is considered one of the most aggressive chemical degradation mechanisms [13,14,15].

The degradation effect of sulfate attack is further exacerbated under wetting–drying (W-D) cycles, during which moisture evaporation promotes sulfate crystallization. The formation of expansive products such as ettringite and gypsum leads to internal stress, microcrack initiation, and deterioration of the interfacial transition zone (ITZ) [16,17,18,19]. These effects are more pronounced in RAC due to its weaker ITZ, higher porosity, and the presence of residual adhered mortar [20,21].

Compared to mechanical strength and durability, the fracture performance of RAC under sulfate attack coupled with W-D cycles has received limited attention [22,23,24]. Crack propagation governs the failure behavior of concrete structures, making it a critical parameter for structural design. In recent years, the advancement of X-ray computed tomography (X-CT) has enabled high-resolution, nondestructive imaging of concrete’s internal microstructure [25,26,27], providing a powerful tool for analyzing crack initiation, expansion, and connectivity evolution [28,29,30].

Moreover, techniques such as digital volume correlation (DVC), fractal dimension analysis [31,32], multiscale porosity assessment [33], and CT-based ITZ layer identification [34] have enabled quantification of parameters like crack volume fraction, connectivity, and porosity. These advances form the basis for linking microstructural damage to macroscopic mechanical performance [35,36,37]. Nevertheless, how to establish predictive models coupling X-CT-derived indicators (e.g., fractal dimension, connectivity index, volume fraction) with fracture parameters such as K_IC and G_F remains largely unexplored [38,39,40,41].

A comprehensive review of the literature reveals that most studies focus on the loss of mass, length change, or compressive strength under sulfate exposure [22,23,35,36,37], while relatively few have investigated the spatial distribution of crack networks and their impact on fracture behavior through quantitative modeling [29,30,40]. In addition, although machine learning methods have been applied to predict degradation trends [38,42], these approaches have rarely incorporated X-CT image features.

In view of this, this paper carried out the sulfate drying and wetting cycle test on recycled concrete and scanned the specimen in situ with X-CT, collected the test scanning results, and analyzed its pore composition. According to the different gray levels of the aggregate, mortar, and pore, it analyzed recycled concrete’s degradation after the sulfate drying and wetting cycle test by using the gray level of the slice level and analyzed its pore change and fracture performance to reveal the recycled concrete meso-damage evolution process. The evolution of internal cracks and fractal dimension in RAC under sulfate wet–dry cycling and its variation with the number of cycles was also studied. The research hypothesis of this study is that the evolution of pore structure induced by wet–dry cycles significantly accelerates the initiation and propagation of cracks, and this process can be characterized by CT grayscale values.

The objective of this study is to investigate the meso-morphology of damage and degradation in recycled concrete subjected to sulfate wet–dry cycles, utilizing X-ray computed tomography (CT) imaging. This study also aims to analyze the cracking behavior of recycled concrete after drying and wetting cycles, with a focus on fracture mechanics. The research further explores the variation in the stress intensity factor (SIF) at the crack tip and determines the critical expansion force for concrete cracking, while assessing the significance of the characteristics of each calculation parameter. The specific research tasks are as follows:

(1) In situ scanning analysis of recycled concrete under various drying and wetting cycles was performed to determine the relationship between the gray scale and the sulfate wet–dry cycle using CT images.

(2) The pore changes and crack propagation in recycled concrete following sulfate wet–dry cycles were analyzed, leading to an understanding of the meso-degradation pattern of the concrete.

(3) The variation in the SIF at the crack tip was examined using fracture mechanics, and the critical expansion force of the recycled concrete was derived. The correlation and significance of various calculation parameters in relation to the critical expansion force were also analyzed.

The structure of this study is organized as follows: Section 2 outlines the principles of CT scanning; Section 3 describes the raw materials and experimental methods, including material sources, performance parameters, testing procedures, and reference standards; Section 4 presents and discusses the experimental results; Section 5 focuses on the fracture behavior of recycled concrete under sulfate-induced dry–wet cycles; and finally, Section 6 summarizes the conclusions of this study and offers engineering recommendations.

## 2. CT Scanning Principle

**Figure 1:**
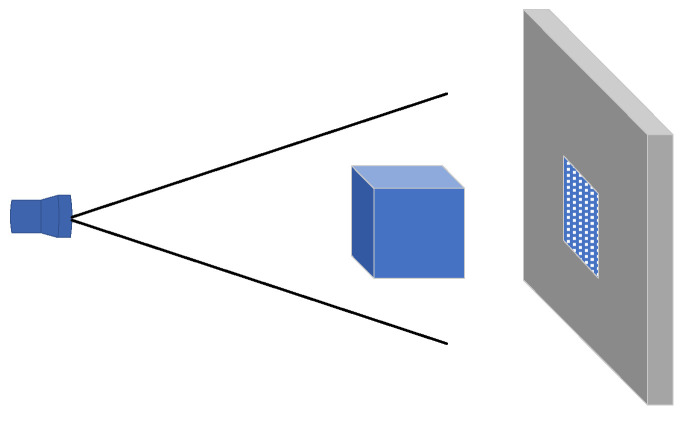
Working principle of CT scanning.

Due to its high image resolution, CT technology is capable of isolating faults detected within an object, preventing interference and distortion, while providing clear and accurate representations of the internal structural relationships, material composition, and defect status of the examined components. X-rays, the most commonly used radiation source in CT devices, are capable of penetrating non-metallic materials. The penetration ability of X-rays varies with different wavelengths, and the absorption capacity of materials for X-rays of the same wavelength also differs. Materials with higher density and higher atomic numbers of constituent atoms exhibit greater X-ray absorption abilities [43]. When X-rays pass through a material, their intensity diminishes according to the Beer–Lambert law, with the degree of attenuation being determined by the material’s linear attenuation coefficient, which is proportional to its density. X-ray computed tomography (X-CT) reconstructs the internal structure of materials by conducting multi-angle scans and calculating the grayscale values of voxels. In recycled concrete, variations in grayscale are directly correlated with the replacement of pore space by sulfate crystals, and changes in the attenuation coefficient indicate crack propagation. CT systems consist primarily of a scanner, a computer system, and an image display and storage system [44], with the operational principle depicted in Figure 1.

## 3. Materials and Methods

**Figure 2:**
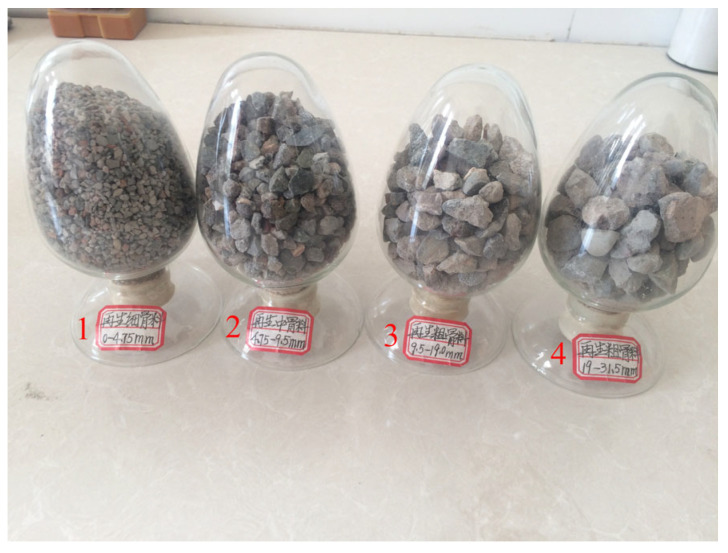
Recycled aggregate. In the figure, 1 denotes recycled fine aggregate; 2 denotes recycled medium aggregate; 3 and 4 denote recycled coarse aggregate.

**Figure 3:**
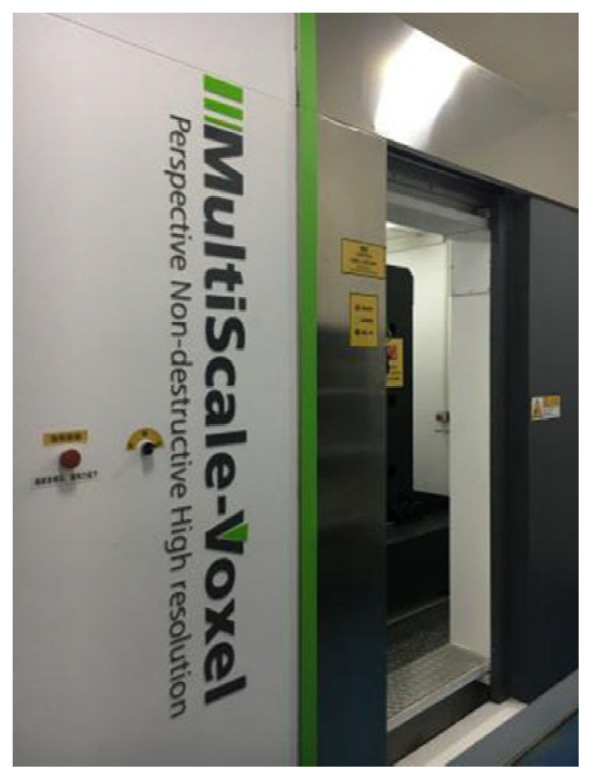
High-precision industrial CT scanning system (Tianjin Sanying Precision Instrument Co., Ltd., Tianjin, China).

**Figure 4:**
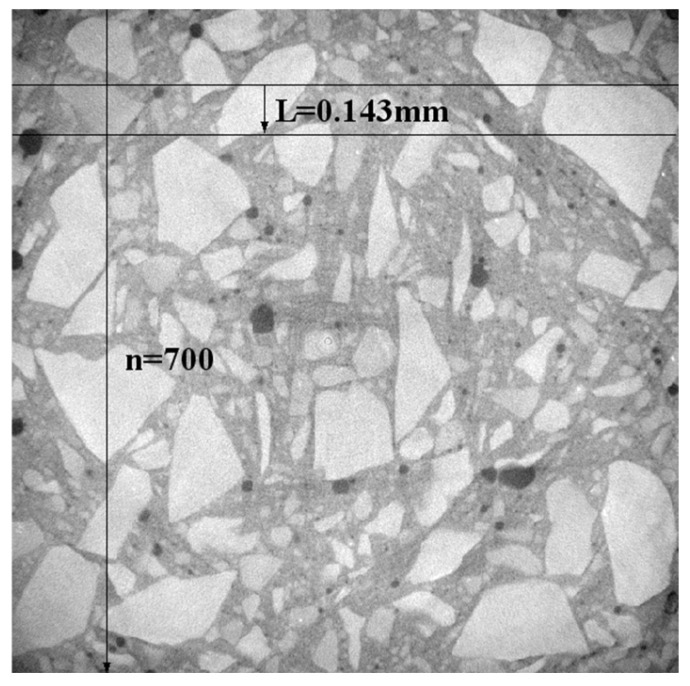
XY section of industrial CT scanning specimen.

The raw materials used in this study are as follows: P42.5 ordinary Portland cement (Gansu Qilian Mountain Cement Group Co., Ltd., Lanzhou, China); crushed stone with a bulk density of 1541 kg/m^3^; medium sand with a bulk density of 1601 kg/m^3^; and homemade recycled aggregate (Figure 2), sourced from demolished C30-grade old concrete, processed through crushing, screening, magnetic separation, and particle grading for sample preparation, with performance parameters detailed in Table 1. Additionally, a 3% content of polycarboxylate superplasticizer was used (Jiangsu Subote New Materials Co., Ltd., Nanjing, China). Recycled concrete specimens with dimensions of 100 mm × 100 mm × 100 mm were prepared according to the mix proportions shown in Table 2. The surface of the prepared specimens was covered with a fresh-keeping film and stored at room temperature for 24 h, after which the formwork was removed. The specimens were then placed in a curing room maintained at a constant temperature of 25–30 °C and relative humidity of 95% for 28 days. Following the curing period, the specimens were removed, placed in an oven, and dried for 1 day. After cooling, the compressive strength of the specimens was measured at 38.35 MPa. The dry density and ultrasonic velocity were measured, and three specimens closest to the average value were selected for CT scanning (Figure 3, Voltage: 110 kV, resolution: 30 μm/voxel). The scanning section spacing was set at 0.143 mm, and 700 layers of images were obtained in one direction, with a total of 2100 layers for the entire specimen (Figure 4).

**Figure 5:**
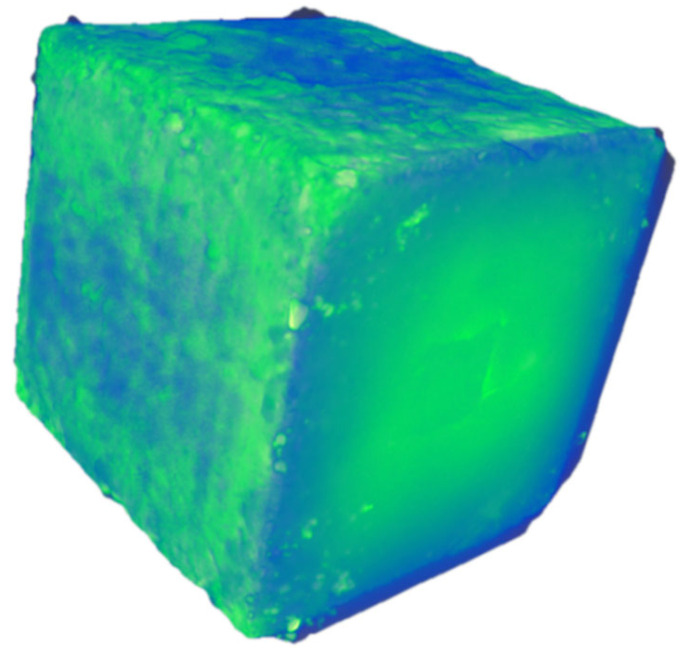
Three dimensional drawing of recycled concrete.

**Figure 6:**
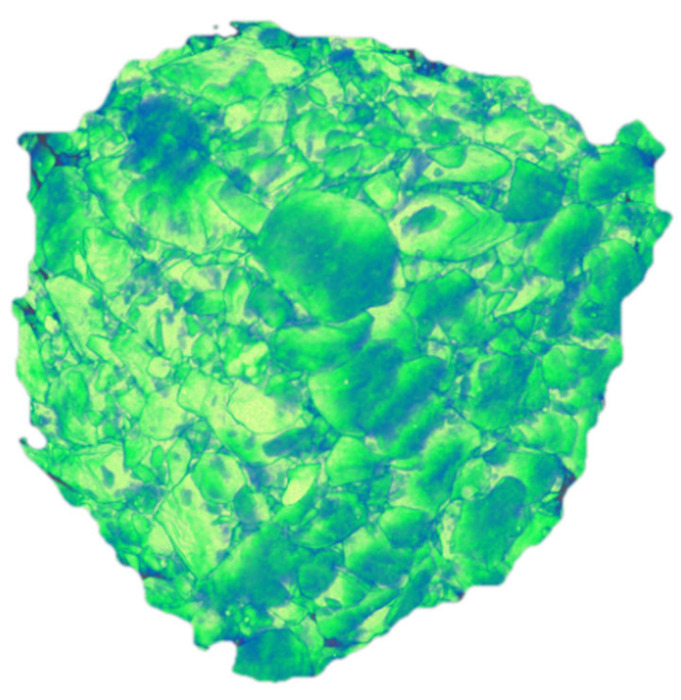
Three dimensional drawing of aggregate.

Three specimens were dried in an oven for 24 h. After cooling, according to ASTM C1012/C1012M-18b [45] and GB/T 50082-2009 [46] standards, a 5% Na_2_SO_4_ solution (optional 10%) is used as the corrosion source, and the solution is replaced every 7 days to maintain a stable concentration. Soak the specimen in Na_2_SO_4_ solution for 15 h (25 ± 2 °C), dry for 9 h, and test various data. The test ended when the crack width on the specimen’s surface was 0.2 mm (according to the restriction standards jointly accepted by EN 1992 [47], ACI 224 [48], and GB 50010 [49]). Four industrial CT scanning tests were carried out during the entire process. Data processing and modeling were carried out with Avizo v. 9.0 software (Figure 5 and Figure 6).

## 4. Results and Discussion

### 4.1. Analysis of Pore Distribution

**Figure 7:**
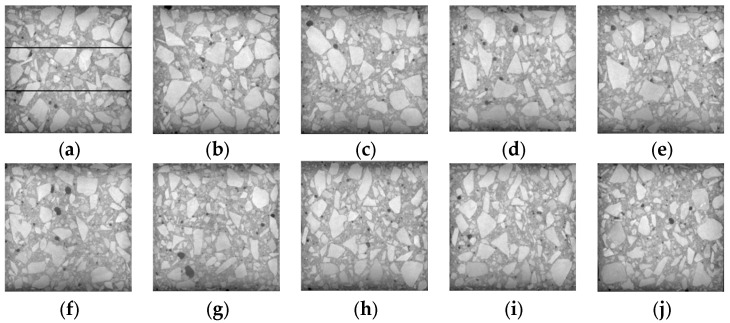
Initial section of specimen. (**b**)~(**j**): Nine arbitrarily selected slices in the XZ plane.

Figure 7 presents the initial section diagram of recycled concrete specimens with different layers. Based on the positional relationship between the upper and lower parts of the specimens in the Z direction, the slices were divided into three layers in the XZ plane (Figure 7a). From top to bottom, the first layer extends from 0 to 34 mm, the second layer from 34 to 77 mm, and the third layer from 77 to 100 mm. Figure 7 illustrates that the lower layer predominantly consists of micropores, the middle layer is characterized by small pores, and the upper layer is dominated by macropores. The results indicate that the internal pores of the specimen are primarily concentrated in the middle and upper layers, with the number and size of pores in these two layers being larger than those in the lower layer. This is attributed to the vibration process, where small-diameter bubbles from the lower layer rise to the middle and upper layers, while medium and large-diameter bubbles break into smaller bubbles, resulting in an increased number of pores in the middle and upper layers after molding. In Figure 7g, the larger pore size in the lower layer may be due to some large-diameter bubbles not breaking during the vibration compaction process, allowing them to rise and form large-aperture pores in the lower layer after specimen formation.

### 4.2. Gray Analysis

**Figure 8:**
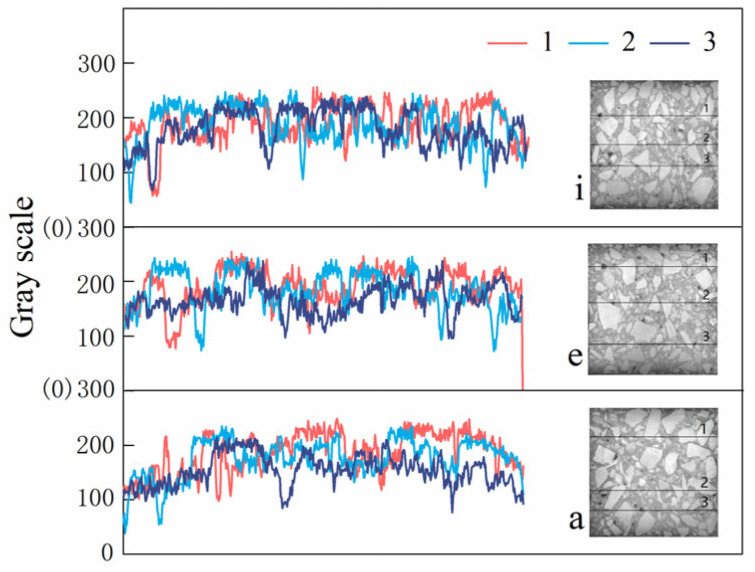
Grayscale image of initial recycled concrete specimen slice line. The subfigures a, e, and i correspond to the subfigures in Figure 7.

Figure 8 presents the grayscale map of the initial recycled concrete specimen slice. The grayscale segmentation threshold was automatically determined using the Otsu algorithm, and image preprocessing was performed with a 5 × 5 median filter to reduce background noise. Three slices from Figure 8 were selected for line gray analysis at three distinct positions. The analysis reveals that the average gray level of the upper layer is the lowest, that of the middle layer is moderate, and that of the lower layer is the highest. Based on the aforementioned stratification (from top to bottom: 0–34 mm, 34–77 mm, and 77–100 mm), these zones are likely indicative of varying sulfate penetration depths, crack propagation regions, or structural damage layers. Such stratified divisions are commonly used in X-CT image analysis to assess the degradation behavior of concrete with respect to depth.

Layer 1 (0–34 mm), located at the top of the specimen and directly exposed to the external environment (e.g., sulfate solution), serves as the primary pathway for ion ingress. This layer is typically associated with sulfate crystallization, crystal expansion-induced cracking, and shrinkage-related cracking [13,14,15,16]. In X-CT images, this region usually shows higher grayscale values, correlating with elevated crack volume fractions and porosity.

Layer 2 (34–77 mm) represents the intermediate zone, dominated by ion diffusion, which may exhibit signs of downward crack propagation and stress transmission from the upper layer. While crack connectivity and sulfate concentration in this layer are lower than in Layer 1, it still plays a critical role in the progression of damage.

Layer 3 (77–100 mm), located near the bottom, typically functions as a structural protection or “control” zone. In the absence of severe external exposure, this layer generally maintains its integrity. However, the presence of through-cracks in this layer suggests substantial degradation, indicating a potential risk of structural instability.

**Figure 9:**
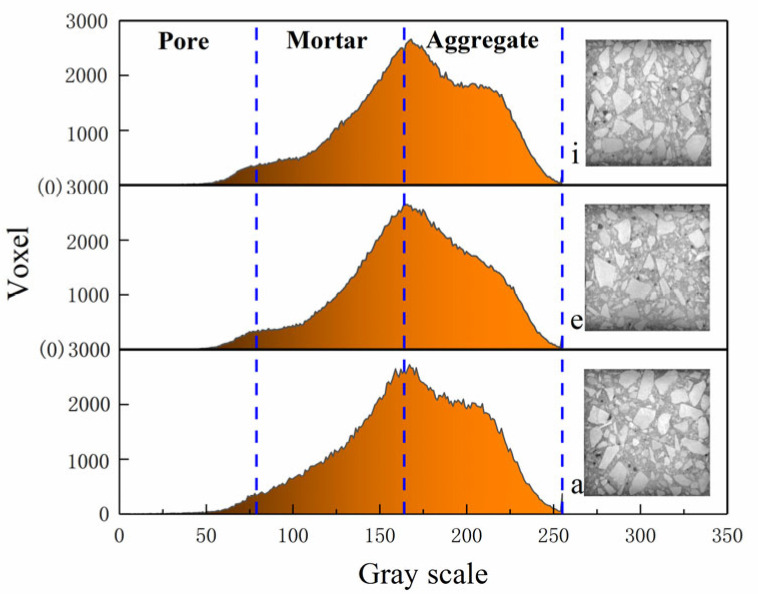
Grayscale image of initial recycled concrete specimen. The subfigures a, e, and i correspond to the subfigures in Figure 7.

**Figure 10:**
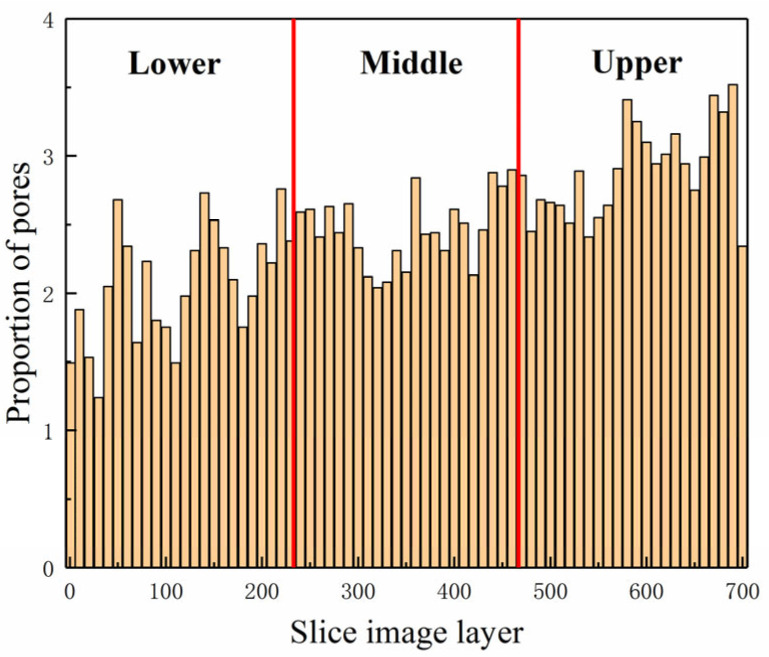
Proportion chart of recycled concrete sample porosity.

The samples consist primarily of aggregate, mortar, and pores, each exhibiting distinct gray levels. The aggregate predominantly appears as bright white and is concentrated in the range of 165–255, the mortar is mostly gray, concentrated in the range of 80–164, and the pores appear dark black, concentrated in the range of 0–79 (Figure 9). Thus, the specimen can be analyzed based on changes in gray levels, and the distribution of porosity in the XY plane is shown in Figure 10. The analysis indicates that the lower layer has the smallest proportion of pores, followed by the middle layer, while the upper layer has the largest proportion. The mean porosity value for the lower layer is 2.07%, for the middle layer is 2.463%, for the upper layer is 2.89%, and for the entire XY plane, it is 2.47%.

**Figure 11:**
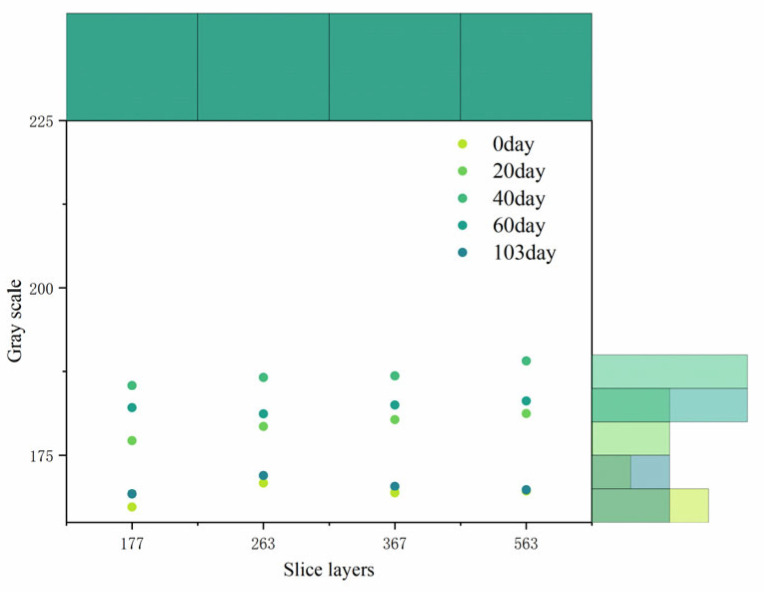
Grayscale change in recycled concrete specimen surface with sulfate drying and wetting cycles.

In this paper, four different slices of the recycled concrete specimens were selected to study the gray changes after the drying and wetting cycles, and Figure 11 shows the gray changes in the specimens after the cycles. As the figure shows, the gray level increased first, and then decreased. When the specimen was not subjected to the drying and wetting cycle, the gray level was approximately 170, and when the drying and wetting cycle was 20~40 d, compared with the initial stage, the gray level of the surface increased by approximately 20%. The reason this occurs is that SO_4_^2−^ ions enter the specimen and react with hydrate to form hydrated calcium silicate to fill part of the pores, which makes the inner part of the specimen more compact, reduces the proportion of pores, and increases the surface gray. After a 40 d drying and wetting cycle, the gray level of the surface began to show a decreasing trend, which was attributable to the continuous filling of the pores until the products expanded and caused cracks in the specimen. This phenomenon lasted until 103 d after the drying and wetting cycle, and the cracks continued to expand until the specimen failed completely (Surface crack width up to 0.2 mm).

### 4.3. Micro Analysis

**Figure 12:**
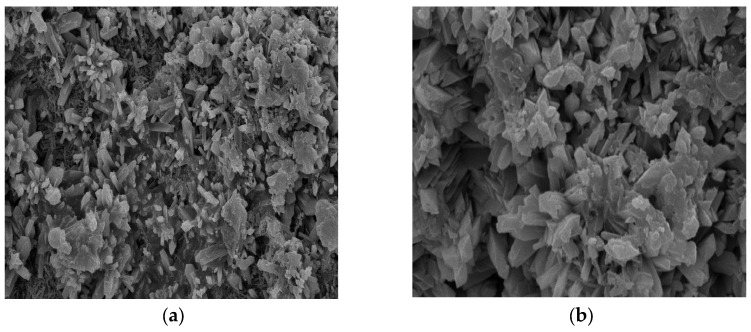
SEM image of recycled concrete after sulfate drying and wetting cycle test. (**a**) 2000 times; (**b**) 5000 times.

**Figure 13:**
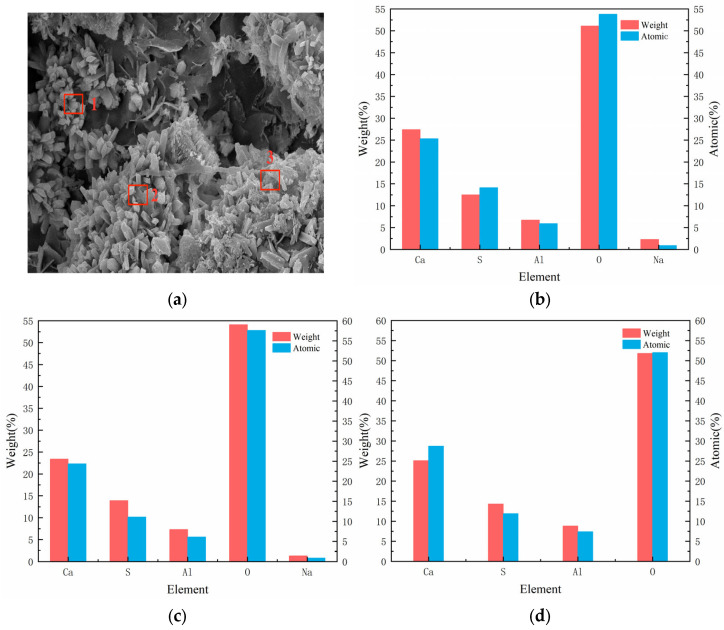
EDS image of recycled concrete after sulfate drying and wetting cycle test. (**a**) Overall picture; (**b**) Spectrum 1; (**c**) Spectrum 2; (**d**) Spectrum 3.

Figure 12 shows the SEM image of recycled concrete after sulfate dry–wet cycling, and Figure 13 shows the corresponding EDS image. By combining Figure 12 and Figure 13, it is evident that the filling phenomenon of sulfate crystals is very prominent. With the infiltration of sulfate, cracks within the concrete expand along the edges of the pores and the interface of the cement matrix. A large amount of sulfate crystals, primarily gypsum and ettringite, were observed to form both inside the cracks and along their edges. These crystals exist in the form of needle-like or plate-like structures, filling the voids of the cracks. Especially at the edges of the pores and the crack tips, the continuous formation of sulfate crystals leads to the expansion of the cracks, further exacerbating the degradation. The sulfate crystallization not only fills the cracks but also generates significant volumetric expansion stress, which promotes further crack propagation. This expansion effect is clearly manifested in the increased crack width and the extended crack paths. As a result of sulfate crystallization, significant changes occurred in the microstructure of the concrete [17,18]. The SEM images clearly show the distribution of these crystals, indicating that sulfate attack not only affects the pore structure of the concrete but also has a profound impact on crack propagation and the overall stability of the material.

### 4.4. Crack Growth Analysis

**Figure 14:**
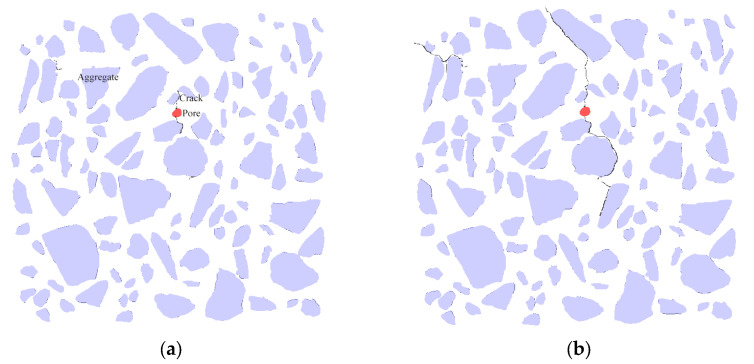
Crack propagation diagram of 177 layers of specimens under different sulfate drying and wetting cycles. (**a**) Cracks after 60 d drying and wetting cycle; (**b**) cracks after 103 d drying and wetting cycle. The red dots indicate pores, and the black curves represent cracks.

**Figure 15:**
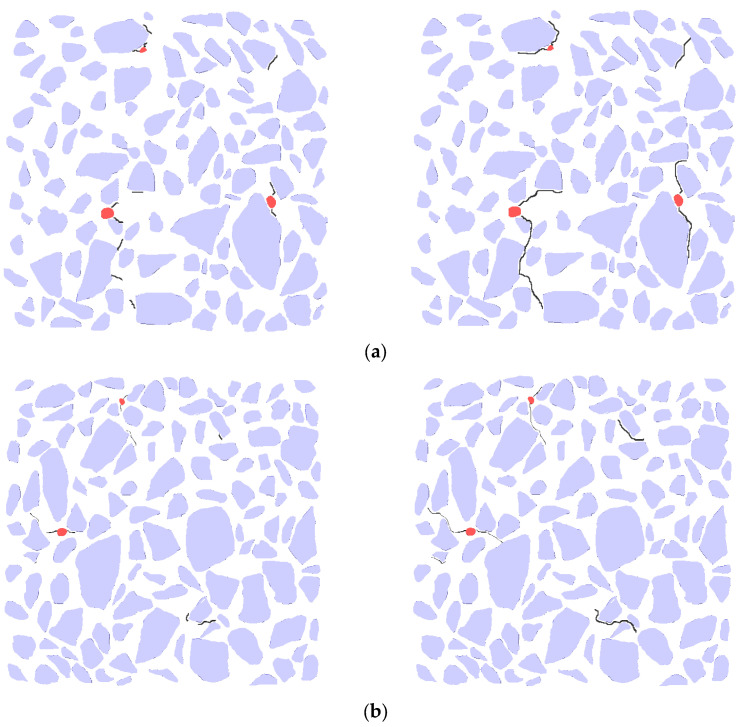

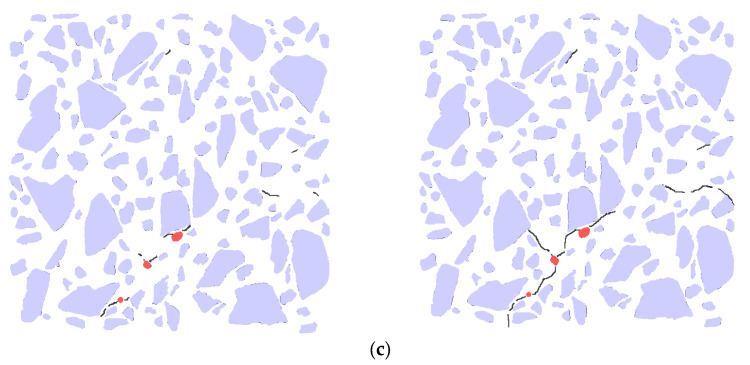
Crack propagation diagram of 177 layers of specimens under different sulfate drying and wetting cycles. (**a**) 263 layer; (**b**) 367 layer; (**c**) 569 layer.

Figure 14 and Figure 15 illustrate the crack propagation diagram of the 177 layers under different sulfate wet–dry cycles. The figure demonstrates that as the wet–dry cycle test progresses, the expansion stress increases, causing the internal weak points of the specimen to crack. Compared to the initial stage, the cracks predominantly appear around the aggregate, particularly in the interface transition zone between the aggregate and mortar. This is because the interface transition zone, which connects the aggregate and mortar, constitutes a part of the internal weak surface of the specimen. Cracks are observed around the harmful pores, which is attributed to the large pore size. As the expansion continues, the expansion stress increases. Although the surrounding areas of macropores may not inherently represent weak points in the specimen, the continuous increase in expansion stress eventually reaches the fracture toughness of the specimen, leading to cracks around these regions. Due to the slight expansion of the pores, dense stress is easily generated around them. After 60 days of the wet–dry cycle, cracks ranging from 3 to 7 mm appeared in the specimen, primarily distributed in the upper and middle layers, which exhibited denser pores. After 103 days of the wet–dry cycle, the internal cracks continued to expand, extending along the interface transition zone, the initial pore positions, and the dense pore areas, until they reached the specimen’s surface. Ultimately, cracks approximately 0.2 mm wide propagated on the surface of the specimen, causing failure. An analysis of the gray level of the cracks showed that they closely resemble the pores, being dark black and concentrated in the range of 0 to 84. The emergence of cracks results in a decrease in the surface gray level, consistent with the findings from previous analyses.

## 5. Fracture Performance Analysis

**Figure 16:**
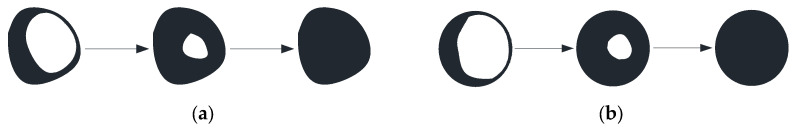
Development diagram of pore expansion in sulfate drying and wetting cycle. (**a**) Development diagram. (**b**) Diagram of expansion development of circular pore.

**Figure 17:**
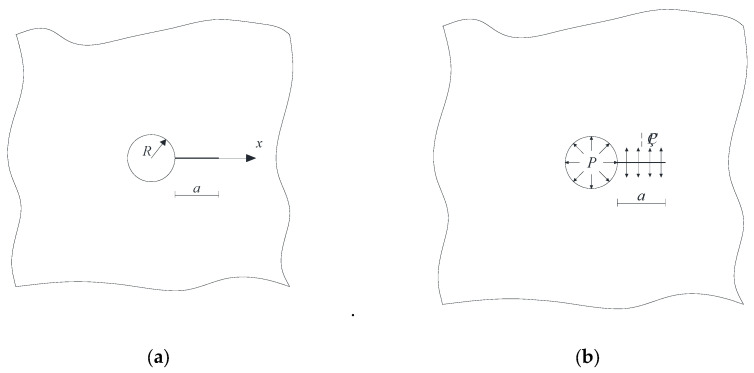
Schematic diagram of a single crack at the edge of the hole. (**a**) Infinite plate with single crack at hole edge; (**b**) Load diagram of single crack at hole edge of infinite plate.

Figure 16a presents a schematic diagram illustrating the development of pore expansion during the sulfate wet–dry cycle. It is evident that under the sulfate wet–dry conditions, the pores primarily fill along the side near the protective layer until the entire pore is completely filled. To facilitate fracture mechanics analysis, this process was modeled as a circular pore diagram, as shown in Figure 16b. During the sulfate wet–dry cycle, a uniform expansion force is generated between the pore and the concrete, leading to the formation of cracks around the pore. Due to the small pore diameter, the cubic concrete specimen can be treated as an infinite plate with a hole edge crack, as depicted in Figure 17a. In the figures, a is the crack length, *R* is the pore radius, and *x* is the coordinate of the horizontal axis along the crack direction. The fracture mechanics analysis of the elastomer is of great significance to the study of the SIF at the crack tip under uniformly distributed load.

### 5.1. Analysis of Single Crack at the Hole Edge

A 100 mm × 100 mm × 100 mm recycled concrete block was used for the sulfate drying and wetting cycle test. The compressive strength of recycled concrete is 38.35 MPa. In the drying and wetting cycle test, the pressure on the hole is the dominant stress, and the load condition of a single crack at the hole’s edge is shown in Figure 17b. According to [50], the calculation formula for the SIF of a single crack at the pore edge of recycled concrete under the sulfate drying and wetting cycle is as follows:

(1) K1=∫0aηP+Pq2×22πa−x1+M11−xa12+M21−xa+M31−xa32dx

**Figure 18:**
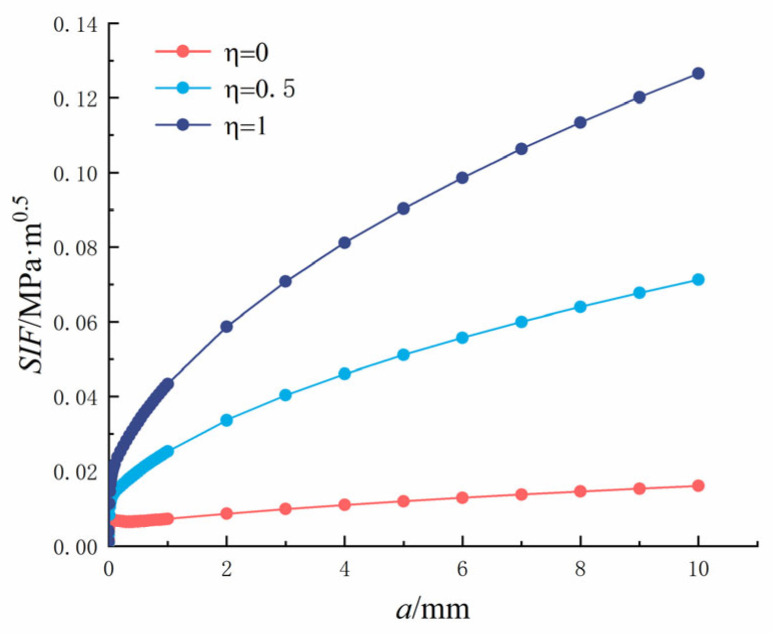
Single crack at the edge of the hole: Relationship between crack length and tip stress intensity factor.

**Figure 19:**
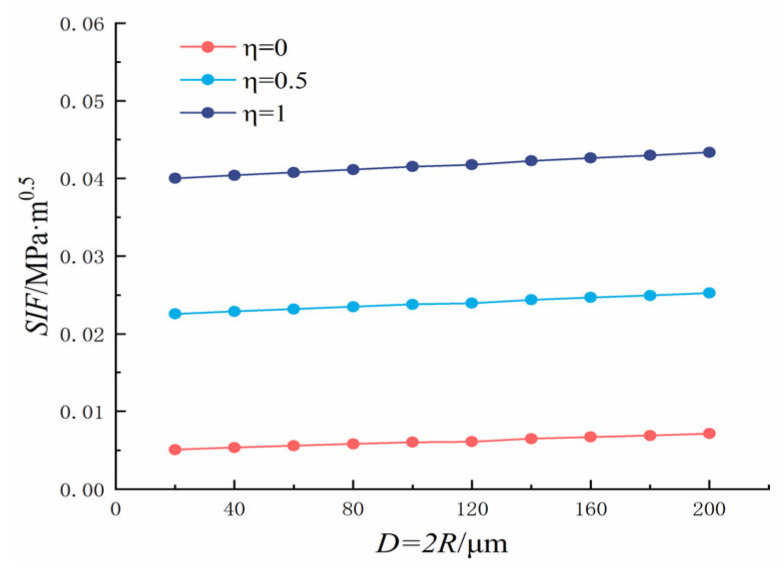
Single crack at the edge of the hole: Relationship between pore diameter and end stress intensity factor.

In which *η* is the ratio of crack to pore stress, 0 ≤ *η* ≤ 1; *K*_1_ is the SIF; *P* is the load; *q* = *R*/(*R* + *x*), and *M*_1_*, M*_2_*, M*_3_ are the parameters of the Glinka Shen analytic weight function. The variation rules of the SIF (*P* = 1) at the crack tip, crack length, and pore diameter are shown in Figure 18 and Figure 19.

It can be seen from Figure 18 that as the crack length increased, the SIF at the crack tip increased. The crack length was between 0 and 1 mm, and it changed rapidly and tended to become stable when it exceeded 1 mm. As *η* increased, the SIF at the crack tip increased gradually and reached the maximum value at *η* = 1. When *η* = 0, the hole is subjected to uniform pressure, and the SIF at the crack tip reached 0.0161 MPa·m^0.5^ when the crack length was 10 mm. When *η* = 0.5, the hole is subjected to uniform pressure, and the SIF at the crack tip reached 0.0713 MPa·m^0.5^ when the crack length was 10 mm. When *η* = 1, the hole is subjected to uniform pressure, and in this case, the SIF at the crack tip reached 0.1265 MPa·m^0.5^ when the crack length was 10 mm.

It can be seen from Figure 19 that as the pore diameter increased, the SIF at the crack tip increased gradually, but only slightly, because of the small change in the pore diameter. As *η* increased, the SIF at the crack tip increased gradually and reached the maximum value at *η* = 1. When *η* = 0, the SIF at the crack tip reached the maximum value of 0.7159 × 10^−^^2^ MPa·m^0.5^ when the pore diameter was 200 μm. When *η* = 0.5, the hole is subjected to uniform pressure, the crack is subjected to half of the reverse symmetric pressure, and the maximum SIF at the crack tip reached 2.5272 × 10^−^^2^ MPa·m^0.5^ when the pore diameter was 200 μm. When *η* = 1, the hole is subjected to uniform pressure, the crack is subjected to the same reverse symmetric pressure as the hole, and the maximum SIF at the crack tip reached 4.3384 × 10^−^^2^ MPa·m^0.5^ when the pore diameter was 200 μm.

According to the fracture criterion, when *K_1_* reaches the fracture toughness of concrete, *K_C_*, the concrete will fracture, and the expansion force generated at this time is its critical expansion force. The conversion relation between concrete’s fracture toughness and compressive strength is [51]:

(2) $$K_{C}=0.06f_{c}^{0.75}$$

**Figure 20:**
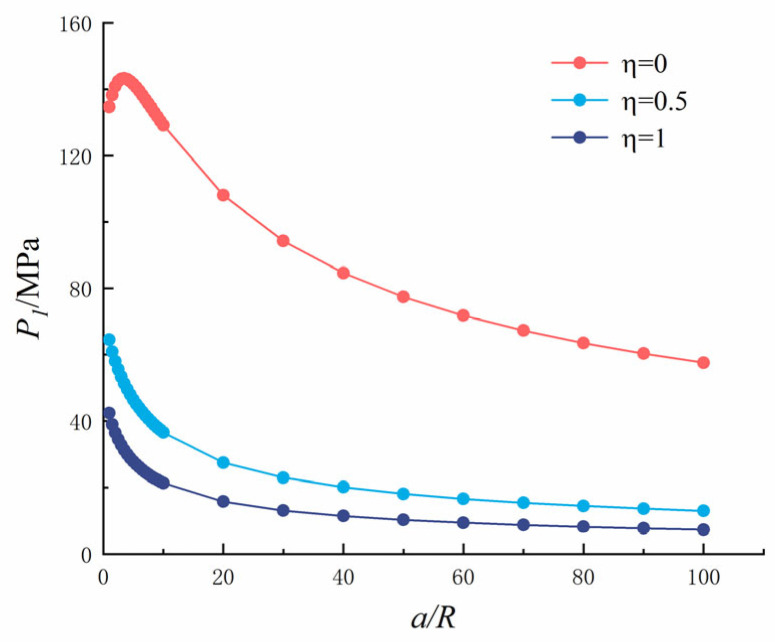
Single crack at the edge of the hole: Relationship between interfacial crack length and critical expansion force.

Let *K*_1_ = *K_C_* and replace Equation (2) with Equation (1); then, the critical expansion force of a single crack in recycled concrete under sulfate expansion can be calculated, as shown in Figure 20.

It can be seen from Figure 20 that as the crack length increased, the critical expansion force decreased gradually and rapidly when *a*/*R* was between 0 and 10, decreased rapidly when *a*/*R* was between 10 and 20, and remained stable when *a*/*R* was between 50 and 100. As *η* increased, the critical expansion force decreased gradually and reached the minimum value at *η* = 1. When *η* = 0, the hole is subjected to uniform pressure, and the critical expansion force reached the maximum value of 134.644 MPa when the crack length was 0 m. When *η* = 0.5, the hole is subjected to uniform pressure, the crack is subjected to half of the reverse symmetric pressure, and the critical expansion force reached the maximum value of 64.506 MPa when the crack length was 0 m. When *η* = 1, the hole is subjected to uniform pressure, the crack is subjected to the same reverse symmetric pressure as the hole, and the critical expansion force reached the maximum value of 42.413 MPa when the crack length was 0 m. As the cracks expand, the expansion force required for concrete to crack becomes smaller and smaller, and it becomes damaged faster. The presence of cracks causes the critical expansion force to decrease, which accelerates the cracking and leads to a decline in the concrete’s durability.

### 5.2. Analysis of Symmetrical Crack at the Hole Edge

**Figure 21:**
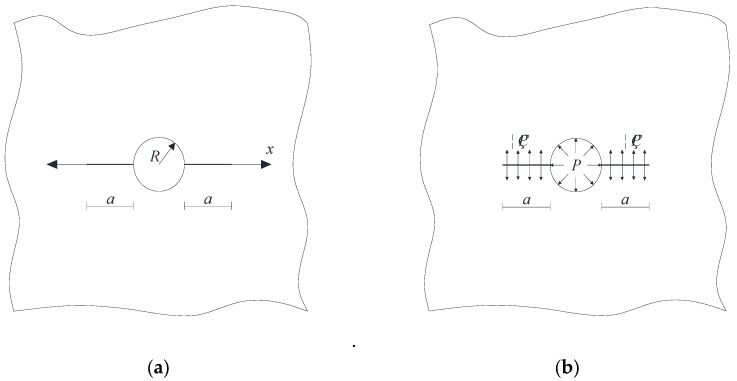
Schematic diagram of symmetrical cracks at the edge of the hole. (**a**) Infinite plates with symmetric cracks on the edge of a hole; (**b**) Load diagram of symmetrical crack at hole edge of infinite plate.

Figure 21b shows the load of symmetrical cracks along the hole’s edge. According to reference [52], the formula to calculate the SIF of a symmetrical crack at the edge of a pore in recycled concrete under the sulfate drying and wetting cycle is as follows:

(3) $$K_{2}=\sigma \sqrt{\pi a}\cdot F_{\eta }\left(s\right)$$

(4) $$F_{\eta }\left(s\right)=\left(1-\eta \right)F_{0}\left(s\right)+\eta F_{1}\left(s\right)$$

In which *σ* is the stress, and *s* = *a*/(*R* + *a*)>, 0 ≤ *s* ≤ 1.

**Figure 22:**
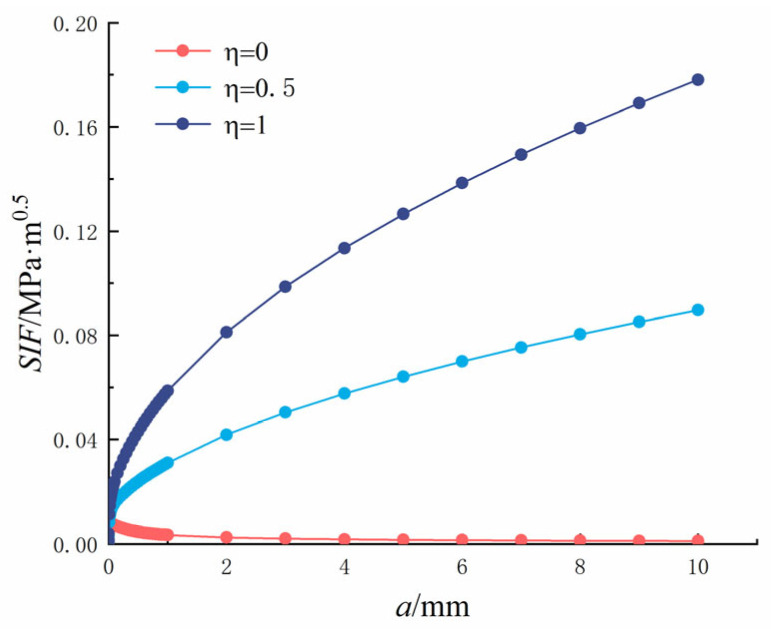
Symmetrical cracks at the edge of the hole: Relationship between crack length and tip stress intensity factor.

**Figure 23:**
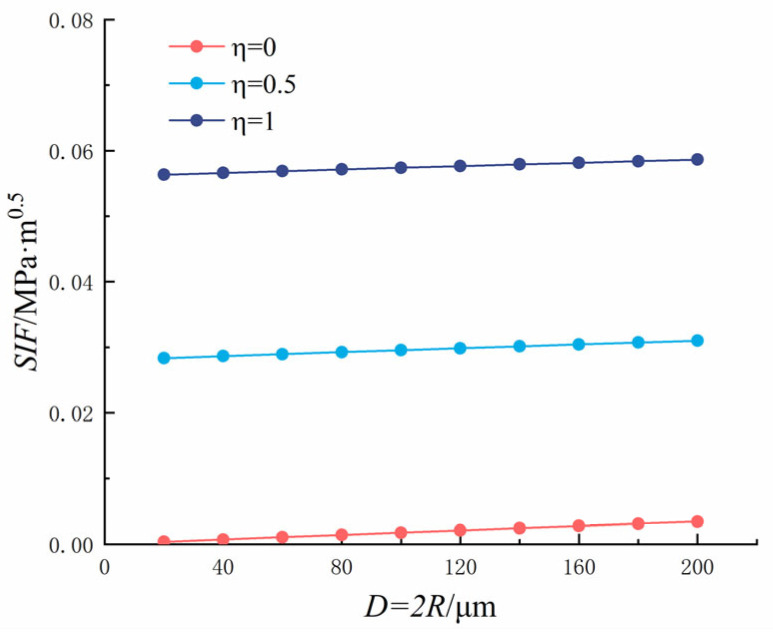
Symmetrical cracks at the edge of the hole: Relationship between pore diameter and end stress intensity factor.

The variation rules of the SIF (*P* = 1) of the crack tip, length, and pore diameter are shown in Figure 22 and Figure 23. As Figure 22 shows, as the crack length increased, the SIF at the crack tip increased, but at *η* = 0, the SIF at the crack tip increased first and then decreased. When the crack length was between 0 and 1 mm, it changed rapidly and tended to become stable when it exceeded 1 mm. As *η* increased, the SIF at the crack tip increased gradually and reached the maximum value at *η* = 1. When *η* = 0, the hole is subjected to uniform pressure, and the SIF at the crack tip reached the maximum value of 0.7393 × 10^−^^2^ MPa·m^0.5^ when the crack length was 0.06 mm. When *η* = 0.5, the hole is subjected to uniform pressure, the crack is subjected to half of the reverse symmetric pressure, and the SIF at the crack tip reached 0.0896 MPa·m^0.5^ when the crack length was 10 mm. When *η* = 1, the hole is subjected to uniform pressure, the crack is subjected to the same reverse symmetric pressure as the hole, and the SIF at the crack tip reached 0.1781 MPa·m^0.5^ when the crack length was 10 mm.

It can be seen from Figure 23 that as the pore diameter increased, the SIF at the crack tip increased gradually, but not greatly because of the small change in pore diameter. As *η* increased, the SIF at the crack tip increased gradually and reached the maximum value at *η* = 1. When *η* = 0, the SIF at the crack tip reached the maximum value of 0.3419 × 10^−^^2^ MPa·m^0.5^ when the pore diameter was 200 μm. When *η* = 0.5, the hole is subjected to uniform pressure, the crack is subjected to half of the reverse symmetric pressure, and the maximum SIF at the crack tip reached 3.1024 × 10^−^^2^ MPa·m^0.5^ when the pore diameter was 200 μm. When *η* = 1, the hole is subjected to uniform pressure, the crack is subjected to the same reverse symmetric pressure as the hole, and the maximum SIF at the crack tip reached 5.8629 × 10^−^^2^ MPa·m^0.5^ when the pore diameter is 200 μm.

**Figure 24:**
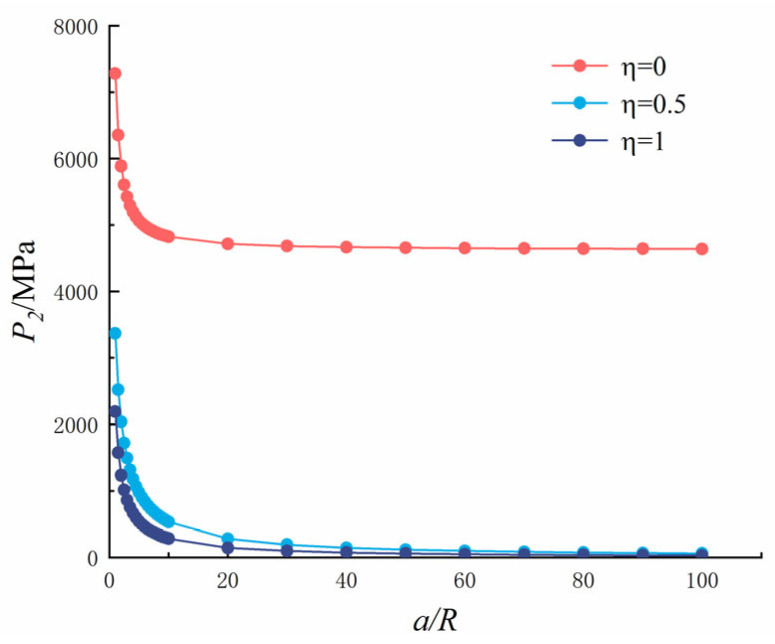
Symmetrical cracks at the edge of the hole: Relationship between interfacial crack length and critical expansion force.

Let *K*_2_ = *K_C_* and replace Equation (2) with Equation (3), and the critical expansion force of a symmetric crack in recycled concrete under sulfate expansion can be calculated, as shown in Figure 24.

It can be seen from Figure 24 that as the crack length increased, the critical expansion force decreased gradually, decreased rapidly when *a*/*R* was between 0 and 10, and when *a*/*R* was between 10 and 20, and then tended to become stable when *a*/*R* was between 30 and 100. As *η* increased, the critical expansion force decreased gradually and reached the minimum value at *η* = 1. When *η* = 0, the hole is subjected to uniform pressure, and the critical expansion force reached the maximum value of 7283 MPa when the crack length was 0 m. When *η* = 0.5, the hole is subjected to uniform pressure, the crack is subjected to half of the reverse symmetric pressure, and the critical expansion force reached the maximum value of 3369 MPa when the crack length was 0 m. When *η* = 1, the hole is subjected to uniform pressure, the crack is subjected to the same reverse symmetric pressure as the hole, and the critical expansion force reached the maximum value of 2192 MPa when the crack length was 0 m. As the cracks expand, the expansion force required to crack the concrete becomes smaller and smaller, the concrete becomes damaged faster, and the presence of cracks causes the critical expansion force to decrease, which accelerates the cracking and reduces the concrete’s durability.

Comparing a single and symmetrical crack, it can be seen that the variation law of SIF is similar, but is not consistent when *η* = 0. Although the change rule of the critical expansion force is consistent, the symmetric crack’s critical expansion force is very large, 53 times greater than that of a single crack. The critical expansion force required for two cracks to occur at the same time is very large, so the probability of symmetrical cracks at the pore’s edge is small, and typically, there is only a single crack at the pore’s edge. As can be seen in the X-CT slice diagram above, there are single, double, and multiple cracks at the pore’s edge, and a single crack appears first. When the expansion force increases further, a second crack (critical expansion force of a single crack) appears at the pore’s edge. As the drying and wetting cycle continues, even third and fourth cracks appear.

### 5.3. Principal Component Analysis

**Figure 25:**
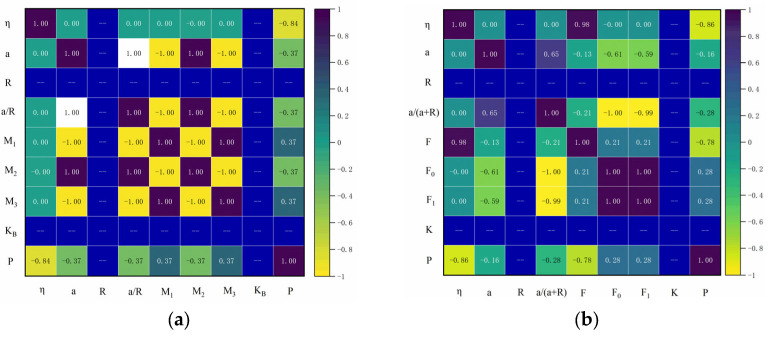
Multiple correlation coefficient matrix of hole edge cracks. (**a**) Single crack at the edge of the hole; (**b**) symmetrical crack at the hole edge.

Figure 25 shows the multiple correlation coefficient matrix for the crack at the pore edge, which illustrates the correlation between the various calculated parameters and the critical expansion force. The darker regions in the figure indicate a higher correlation between input factors and output results, while the lighter regions indicate a lower correlation. A positive correlation value indicates a positive correlation between the two, while a negative value indicates a negative correlation [53].

From Figure 25, it can be seen that η has a high negative correlation with the critical expansion force. For a single crack at the pore edge, the correlation coefficient between η and the critical expansion force is −0.84, and for a symmetric crack at the pore edge, the correlation coefficient is −0.86. From Figure 25a, it can be seen that the correlation coefficients of a, a/R, and M2 with the critical expansion force are −0.37, and the correlation coefficients of M2 and M3 with the critical expansion force are 0.37. From Figure 25b, it can be seen that F also has a high negative correlation with the critical expansion force, with a correlation coefficient of −0.78. The correlation coefficients of the remaining parameters with the critical expansion force are all less than 0.3.

**Figure 26:**
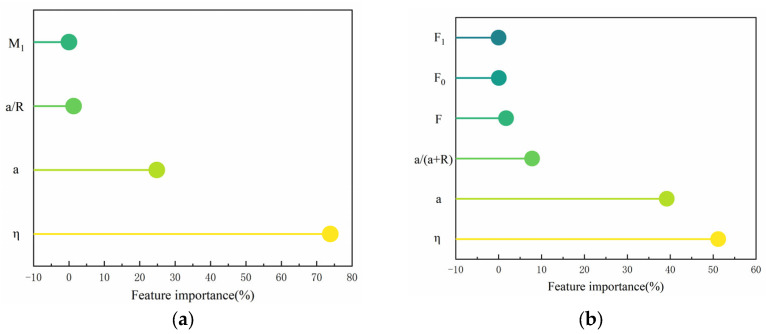
Feature importance analysis. (**a**) Single crack at the edge of the hole; (**b**) symmetrical crack at the hole edge.

The mutual variations between the calculated parameters exhibit different degrees of significance. To investigate the impact of these parameters on the critical expansion force and perform a quantitative analysis of each influence, principal component analysis (PCA) was employed [54]. PCA explores quantitative data and condenses it into several aspects (principal components), as shown in the extraction of principal components in Figure 26.

Figure 26 demonstrates the importance of each parameter in influencing the critical expansion force. It can be observed that η and a have the greatest influence on the critical expansion force, while the remaining parameters have a smaller impact. This is consistent with the calculation results. In this study, more complex conditions were not considered; however, in real-world scenarios, the critical expansion force might vary under more complex conditions, which could lead to a change in the ranking of feature importance.

## 6. Conclusions and Recommendations

In this study, using the CT images of recycled concrete under the sulfate drying and wetting cycle, the change rule of the gray level, and the meso-morphology of recycled concrete’s degradation were obtained. Combined with fracture mechanics, the cracking problem of recycled concrete after the drying and wetting cycles was analyzed. The variation law of the SIF at the crack tip was analyzed, and the relation between the critical expansion force and crack length was discussed. Based on the discussion above, the following conclusions can be drawn:

(1) Pore Distribution and Material Characterization. The analysis revealed that the pores were primarily concentrated in the middle and upper layers of the concrete. The gray levels of the aggregate, mortar, and pores exhibited distinct differences, with the aggregate appearing predominantly bright white (gray levels between 165 and 255), the mortar being gray, and the pores being dark black. These variations in gray levels effectively differentiated the three material phases in the concrete.
(2) Impact of Dry–Wet Cycling on Pore Structure and Crack Formation. The dry–wet cycle tests demonstrated that both the number and size of pores decreased, while expansion stress increased. Cracks appeared first in the internal interface transition zone around large pores and within dense small pores. The evolution of the pore structure and the propagation of cracks were consistent with the changes in the gray levels observed during X-CT scanning. This suggests that monitoring gray level variation is a reliable and effective method for assessing the degradation of RAC following sulfate dry–wet cycling.
(3) Crack Propagation and Stress Intensity Factors (SIFs). As crack length increased, the Stress Intensity Factor (SIF) of both single cracks and symmetric crack tips showed an upward trend. Similarly, the SIF increased with pore diameter, although the change was relatively small. Furthermore, as the crack length increased, the critical expansion force gradually decreased. Notably, the critical expansion force at the symmetric crack tip near the pore edge was 53 times greater than that of a single crack, highlighting the significant role of pore edges in crack resistance.

In conclusion, the results of this study provide valuable insights into the degradation mechanisms of recycled aggregate concrete under sulfate dry–wet cycling. The findings suggest that X-CT-based analysis is a powerful tool for monitoring material deterioration; the research hypothesis of this study is valid. The internal expansion force of the pores caused by the wet–dry cycles continues to increase until the critical expansion force is reached, at which point cracks form at the pore edges. As the experiment progresses, these cracks gradually expand. This process can be characterized by the CT grayscale values. However, the current study has certain limitations, such as the spatial resolution of CT and the variability in the sources of recycled aggregates. In future research, multi-scale image processing could be introduced, and the performance evolution analysis could be expanded to include multi-environmental coupled effects, and further quantitatively analyze the crack development rate under the drying and wetting cycle of sulfate.

## Figures and Tables

**Table 1:** Performance indicators of recycled coarse aggregate.

Water Absorption Rate (%)	Mud Content (%)	Crushing Index (%)	Bulk Density (%)	Apparent Density (kg/m^3^)
2.2	0.5	9.6	1488	2452

**Table 2:** Mix proportion of recycled concrete; unit: kg/m^3^.

Cement	Coarse Aggregate	Fine Aggregate	Recycled Coarse Aggregate	Water Reducer	Water
424	933.6	614	233.4	12.72	180

## Data Availability

The raw data supporting the conclusions of this article will be made available by the authors on request. Due to concerns regarding data privacy, and the fact that some datasets are still under further analysis, the complete data underlying this study are not publicly available. However, all relevant data can be provided upon reasonable request to the corresponding author.
